# Identification of the shared genes and immune signatures between systemic lupus erythematosus and idiopathic pulmonary fibrosis

**DOI:** 10.1186/s41065-023-00270-3

**Published:** 2023-03-04

**Authors:** Sheng Liao, Youzhou Tang, Ying Zhang, Qingtai Cao, Linyong Xu, Quan Zhuang

**Affiliations:** 1 Transplantation Center, the 3rd Xiangya Hospital, Central South University, 138 Tongzipo Rd, Changsha, 410013 Hunan China; 2 Department of Nephropathy, the 3rd Xiangya Hospital, Central South University, Changsha, China; 3 School of Life Science, Central South University, Changsha, China; 4 Research Center of National Health Ministry on Transplantation Medicine, 138 Tongzipo Rd, Changsha, 410013 Hunan China

**Keywords:** Systemic lupus erythematosus, Pulmonary fibrosis, MAPK, Immune cell, microRNA

## Abstract

**Background:**

Systemic lupus erythematosus (SLE) is an autoimmune disorder which could lead to inflammation and fibrosis in various organs. Pulmonary fibrosis is a severe complication in patients with SLE. Nonetheless, SLE-derived pulmonary fibrosis has unknown pathogenesis. Of pulmonary fibrosis, Idiopathic pulmonary fibrosis (IPF) is a typicality and deadly form. Aiming to investigate the gene signatures and possible immune mechanisms in SLE-derived pulmonary fibrosis, we explored common characters between SLE and IPF from Gene Expression Omnibus (GEO) database.

**Results:**

We employed the weighted gene co-expression network analysis (WGCNA) to identify the shared genes. Two modules were significantly identified in both SLE and IPF, respectively. The overlapped 40 genes were selected out for further analysis. The GO enrichment analysis of shared genes between SLE and IPF was performed with ClueGO and indicated that p38MAPK cascade, a key inflammation response pathway, may be a common feature in both SLE and IPF. The validation datasets also illustrated this point. The enrichment analysis of common miRNAs was obtained from the Human microRNA Disease Database (HMDD) and the enrichment analysis with the DIANA tools also indicated that MAPK pathways’ role in the pathogenesis of SLE and IPF. The target genes of these common miRNAs were identified by the TargetScan7.2 and a common miRNAs-mRNAs network was constructed with the overlapped genes in target and shared genes to show the regulated target of SLE-derived pulmonary fibrosis. The result of CIBERSORT showed decreased regulatory T cells (Tregs), naïve CD4+ T cells and rest mast cells but increased activated NK cells and activated mast cells in both SLE and IPF. The target genes of cyclophosphamide were also obtained from the Drug Repurposing Hub and had an interaction with the common gene PTGS2 predicted with protein-protein interaction (PPI) and molecular docking, indicating its potential treatment effect.

**Conclusions:**

This study originally uncovered the MAPK pathway, and the infiltration of some immune-cell subsets might be pivotal factors for pulmonary fibrosis complication in SLE, which could be used as potentially therapeutic targets. The cyclophosphamide may treat SLE-derived pulmonary fibrosis through interaction with PTGS2, which could be activated by p38MAPK.

**Supplementary Information:**

The online version contains supplementary material available at 10.1186/s41065-023-00270-3.

## Introduction

Systemic lupus erythematosus (SLE) is a condition in which the immune system attacks the healthy body due to the exaggerated B cell and T cell responses and loss of immune tolerance against self-antigens. Circulated immune complex and activated complements and cytokines lead to clinical manifestations of SLE, from mild fatigue to organ damage [1]. These immune complex and cytokines may deposit in lung, causing pulmonary involvement. The pulmonary manifestations of the disease include pulmonary hemorrhage, pulmonary hypertension and interstitial lung disease [2, 3]. Despite the morbidity is less than 15%, interstitial lung disease can result in very poor prognosis and outcomes in SLE patients [4]. Pulmonary fibrosis, a development of interstitial lung disease, decreases the pulmonary function and have a significant impact on the quality of patients’ life. Recently, some reports displayed pulmonary fibrosis might be more common than that has been previously thought in SLE patients [5–7]. Additionally, the identified CT morphologic characteristics indicated the distinctive pulmonary fibrosis pattern in SLE patients [3]. At present, the association between some cytokines and pulmonary fibrosis in SLE have been demonstrated, such as CXCL10, CXCL11, IL-8 and so on [8–10]. However, research about the underlying mechanism is still lacking, especially at the gene level.

Idiopathic pulmonary fibrosis (IPF) is a typicality and lethal form of pulmonary fibrosis, with poor prognosis and unclear pathogenesis [11]. Since the dataset about pulmonary fibrosis secondary to SLE is still rare and a considerable part of IPF patients would be owed to SLE as disease progressed and more symptoms appeared [12, 13], we chose IPF and SLE dataset to try to explore the pathogenesis in SLE-derived pulmonary fibrosis. We tried to use the weighted gene co-expression network analysis (WGCNA) to identify the gene clusters of related and connected shared genes in SLE and IPF. We identified the co-expression modules in SLE and IPF using the published gene expression data from the Gene Expression Omnibus (GEO) (http://www.ncbi.nlm.nih.gov/geo/). We explored interaction and the potential treatment effect of cyclophosphamide. We also evaluated the state of infiltrating immune cells in SLE and IPF. Our results revealed that p38MAPK cascade and the infiltration of some immune-cell subsets, such as Tregs, naïve CD4+ T cells, activated NK cells and mast cells, are associated with the progress of SLE-derived pulmonary fibrosis. And the target genes of cyclophosphamide had an interaction with the common gene PTGS2. Our study demonstrated a possible mechanism of SLE-derived pulmonary fibrosis. These may contribute to the management of pulmonary fibrosis in SLE patients and improve SLE patients’ life quality.

## Materials & methods

### GEO dataset download and process

We used the key word “system lupus erythematosus” and “idiopathic pulmonary fibrosis” to search for SLE and IPF gene expression profiles in the GEO database respectively. The following criteria filter the obtained dataset: First, the gene expression profiling must include cases and controls. Second, the organization used for sequencing should be peripheral blood mononuclear cells (PBMCs). Third, the number of samples in each group should not be less than 10 to ensure the accuracy of the WGCNA. Fourth, these datasets must provide the processed data or raw data that could be used for re-analyzation. Finally, the GEO dataset numbered GSE50772 and GSE28042 were selected. The normalized Series Matrix Files were provided by the contributors and log2 transform was performed for gene expression profiling when needed. Then, the probes to the gene symbols were matched according to the annotation document of corresponding platforms. Finally, the gene matrix was obtained. The GSE50772 and GSE28042 were paired as a discovery cohort for the WGCNA analysis and subsequent analyses. Their detailed information was summarized in Table 1. In addition, GSE154851 and GSE33566 were used for validation (Table 2).

**Table 1:** Summary of two GEO datasets involving SLE and IPF patients

GSE number	Platform	Samples	Source types	Disease
GSE50772	GPL570	61 patients and 20 controls	PBMC	SLE
GSE28042	GPL6480	75 patients and 19 controls	PBMC	IPF

**Table 2:** Summary of two validated datasets involving SLE and IPF patients

GSE number	Platform	Samples	Source types	Disease
GSE154851	GPL16699	38 patients and 32 controls	Peripheral blood	SLE
GSE33566	GPL6480	93 patients and 30 controls	peripheral blood	IPF

### Weighted gene co-expression network analysis

The weighted gene co-expression network analysis (WGCNA) is an algorithm which is able to find the co-expressed gene modules with high biological significance and explore the relationship between gene networks and diseases [14]. We used the WGCNA to obtain the SLE and IPF associated modules. More than 20,000 genes were obtained by sequencing in the GEO dataset, and the top 10,000 genes were selected according to the MAD for the WGCNA analysis. The “WGCNA” package in R.4.0.5 software were used to perform the WGCNA analysis. The appropriate soft powers β (ranged from 1 to 20) was selected using the function of “pickSoftThreshold” in the WGCNA package according to the standard of scale-free network. Next, the soft power value β and gene correlations matrix among all gene pairs calculated by Pearson analysis were used to build adjacency matrix, which was calculated by the formula: *a*_*ij*_ = |*S*_*ij*_|^*β*^ ( *a*_*ij*_: adjacency matrix between gene i and gene j, *S*_*ij*_: similarity matrix which is composed of Pearson correlation coefficients of all gene pairs, β: soft power value). Then the topological overlap matrix (TOM) and the corresponding dissimilarity (1 − TOM) was transformed from the adjacency matrix. A hierarchical clustering dendrogram was further built and similar gene expressions were divided into different modules. Finally, the expression profiles of each module were summarized by the module eigengene (ME) and the correlation between the ME and clinical features was calculated. The modules with high correlation coefficient with clinical features were focused and the genes in these modules were selected for further analyses. In this study, the soft threshold β was chosen 3 in the WGCNA analysis of SLE and 5 in IPF. The other parameters were default.

### Identification of shared gene signatures in SLE and IPF

We selected the modules that were highly related to SLE and IPF. The shared genes in modules positively associated with SLE and IPF were overlapped using VennDiagram package. ClueGO is a Cytoscape plug-in, which could categorize the non-redundant GO terms and visualize them as a functionally grouped network [15]. To explore potential roles of these shared genes in SLE and IPF, a biological analysis of these shared genes was performed with ClueGO. The biological process of GO analysis was focused. The *p*-value < 0.05 was considered significant. The PPI network also was made with Cytoscape to find the top genes which might be more important in the pathogenesis.

### Identified the common MicroRNAs in SLE and IPF

MicroRNAs (miRNAs), a kind of small non-coding RNAs, have been demonstrated to regulate gene expression by promoting mRNA degradation or inhibiting mRNA translation. Therefore, we tried to explore whether some miRNAs are regulating these risk genes in SLE and IPF. The Human microRNA Disease Database (HMDD) is a database that curated experiment-supported evidence for human miRNA and disease associations [16]. The SLE-associated and IPF-associated miRNAs were obtained and took an intersection of them. Then we further identified the expression situation of these miRNAs in SLE and IPF based on published literature according to the HMDD, and only miRNAs with the same disorder types were further analyzed. Finally, we perform GO biological processes analysis with the mirPath v3.0 software in DIANA tool to explore the function of common miRNA. The GO terms with *p*-values < 0.01 were considered significant.

### The construction of common miRNAs-target genes network

TargetScan (http://www.targetscan.org) predicts biological targets of miRNAs by searching for the presence of conserved 8mer, 7mer, and 6mer sites that match the seed region of each miRNA. The intersection of target shared-genes of common miRNAs in SLE and IPF were used to construct the miRNAs–mRNAs regulated network. The visualized network was built with Cytoscape. HALLMARK_HYPOXIA gene set from Molecular Signatures Database (https://www.gsea-msigdb.org/gsea/msigdb/index.jsp) includes genes up-regulated in response to hypoxia. We used this gene set to identify the hypoxia-related genes in our common miRNAs-target genes network due to the strong relationship between hypoxia-related genes and the pulmonary fibrosis.

### The relationship between genes and infiltrating immune cells

CIBERSORT is an algorithm to impute gene expression profiles and provide an estimation of the abundances of member cell types in a mixed cell population, using gene expression data [17]. In our research, we used the CIBERSORT to get the state of infiltrating immune cells in patient with SLE or IPF. Then, we explored the relationship between the top genes and the shared genes that were regulated by the common miRNAs and the infiltrating immune cells.

### Analysis of target genes of cyclophosphamide treatment

The target gene of cyclophosphamide was obtained from the Drug Repurposing Hub (https://www.broadinstitute.org/drug-repurposing-hub). Subsequently, we constructed a PPI network comprised target genes and the top10 genes associated with mechanism between SLE and IPF.

### Gene set enrichment analysis

Gene Set Enrichment Analysis (GSEA) is a computational method that determines whether an a priori defined set of genes shows statistically significant, concordant differences between two biological states [18]. R.4.0.5 software and related packages were used.

### Analysis of molecular docking

To analyze the binding affinities and modes of interaction between the drug and targets, AutodockVina 1.2.2, a silico protein–ligand docking software was employed [19]. The molecular structures of cyclophosphamide were retrieved from PubChem Compound (https://pubchem.ncbi.nlm.nih.gov/) [20]. The 3D coordinates of PTGS2 (PDB ID, 5F19; resolution, 2.04 Å) and CYP2C19 (PDB ID, 4GQS; resolution, 2.87 Å) were downloaded from the PDB (http://www.rcsb.org/pdb/home/home.do). Rigid protein–protein docking (ZDOCK) was performed between PTGS2 and CYP2C19 to study the relationships. The ZDOCK module was run to identify the docking sites and calculate the ZDOCK scores.

## Results

### The co-expression modules in SLE and IPF

**Fig. 1:**
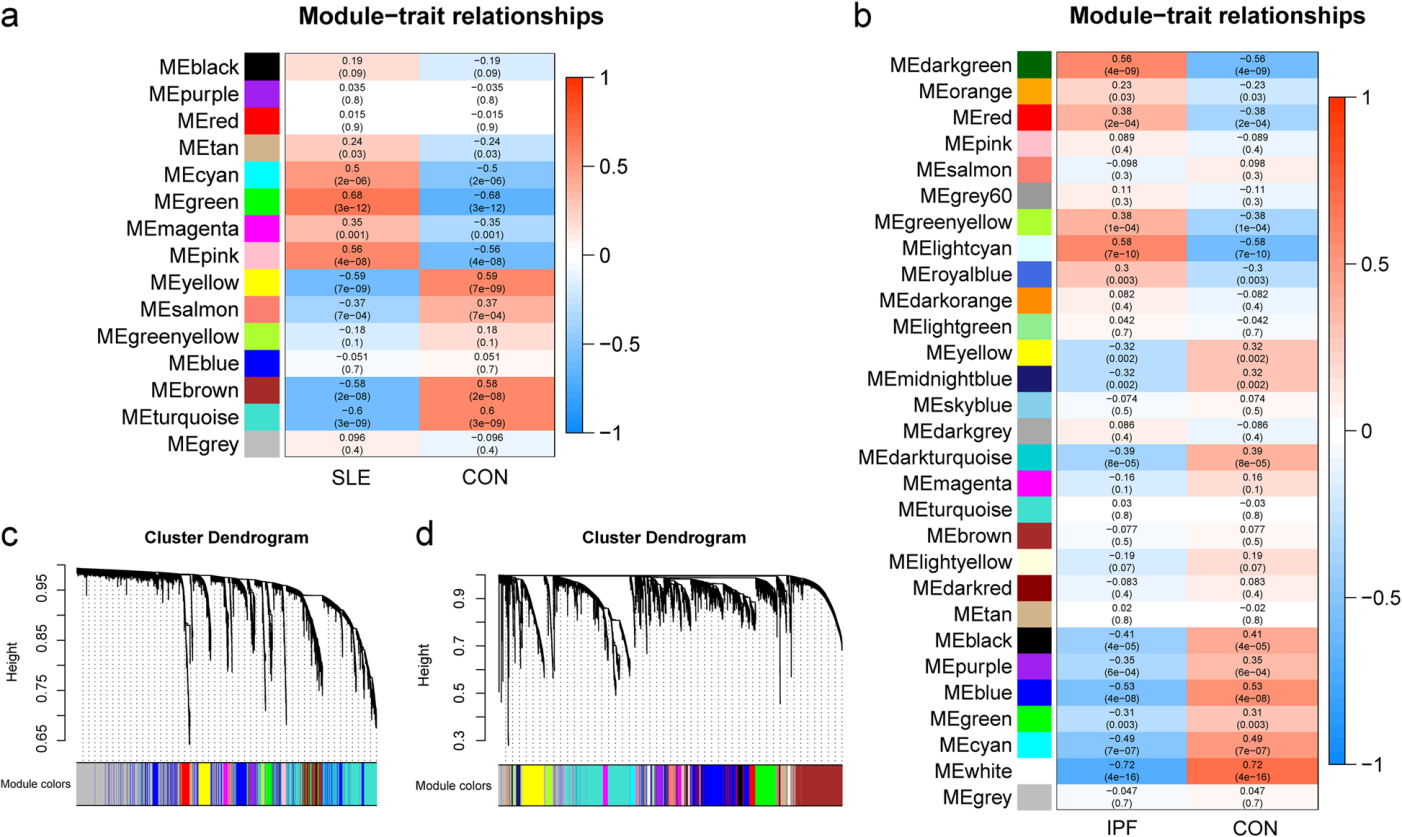
Weighted gene co-expression network analysis (WGCNA). **a** Module–trait relationships in SLE. The corresponding correlation and *p*-value are exhibited in each cell. **b** Module–trait relationships in IPF. The corresponding correlation and p-value are exhibited in each cell. **c** The cluster dendrogram of co-expression genes in SLE. **d** The cluster dendrogram of co-expression genes in IPF

Totally, 15 modules were identified in GSE50772 through the WGCNA, in which different colors represented different modules. Following, a heat map about module-trait relationships was mapped according to the Pearson correlation coefficient to evaluate the association between each module and the disease. Two modules with high association with SLE were selected as the SLE-related modules (green: r = 0.68, *p* = 3e-12; pink: r = 0.56, *p* = 4e-08). The green and pink modules, including 330 and 152 genes respectively, were positively correlated with SLE (Fig. 1a, c). Also, 29 modules were identified in GSE28042, in which the darkgreen and lightcyan modules were highly positively associated with IPF (darkgreen: r = 0.56, p = 4e-09; lightcyan: r = 0.58, *p* = 7e-10), including 54 and 97 genes respectively (Fig. 1b, d).

### The common gene signatures in SLE and IPF

**Fig. 2:**
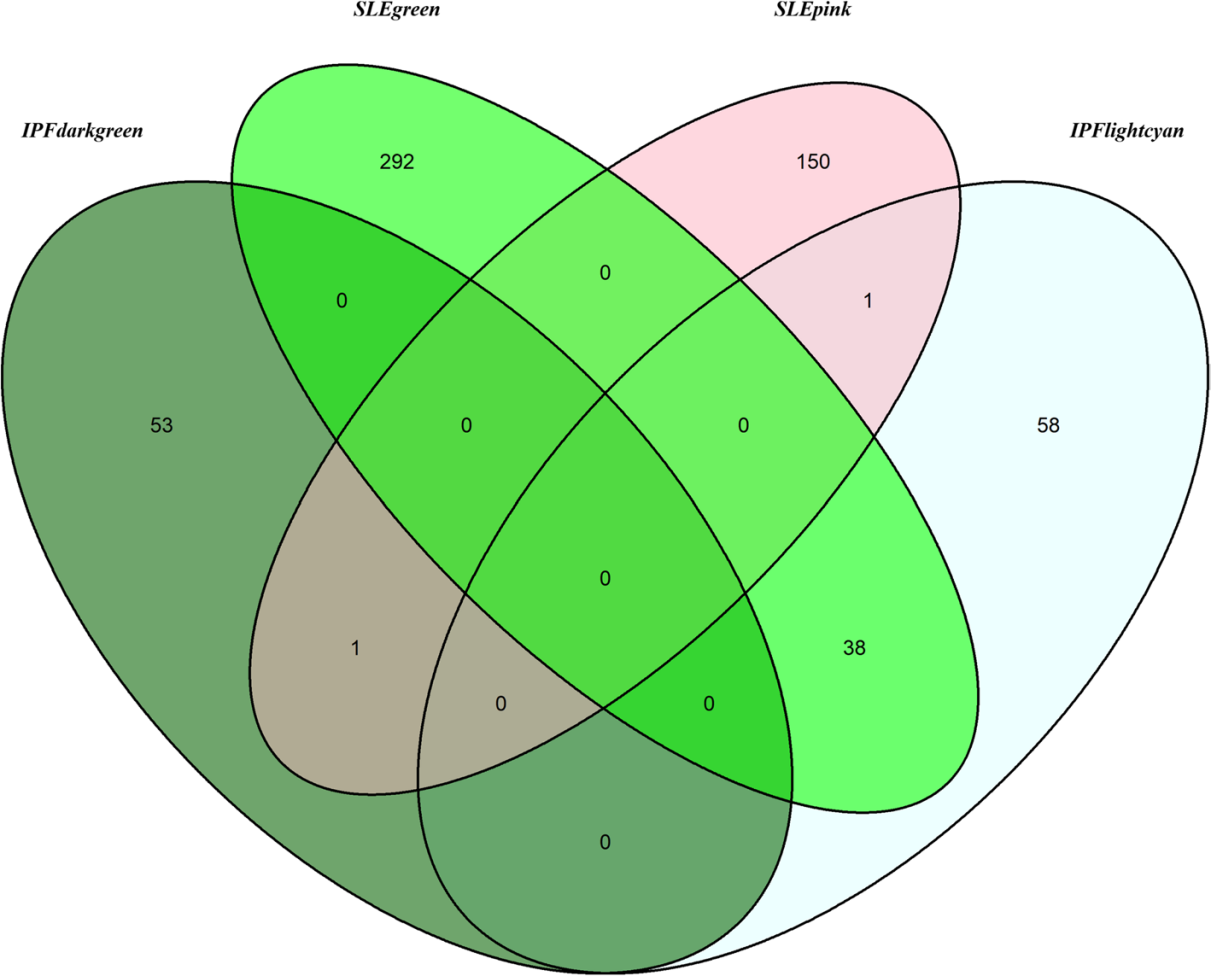
The shared genes between the green and pink modules of SLE and the darkgreen and lightcyan modules of IPF, identified in the overlap

**Fig. 3:**
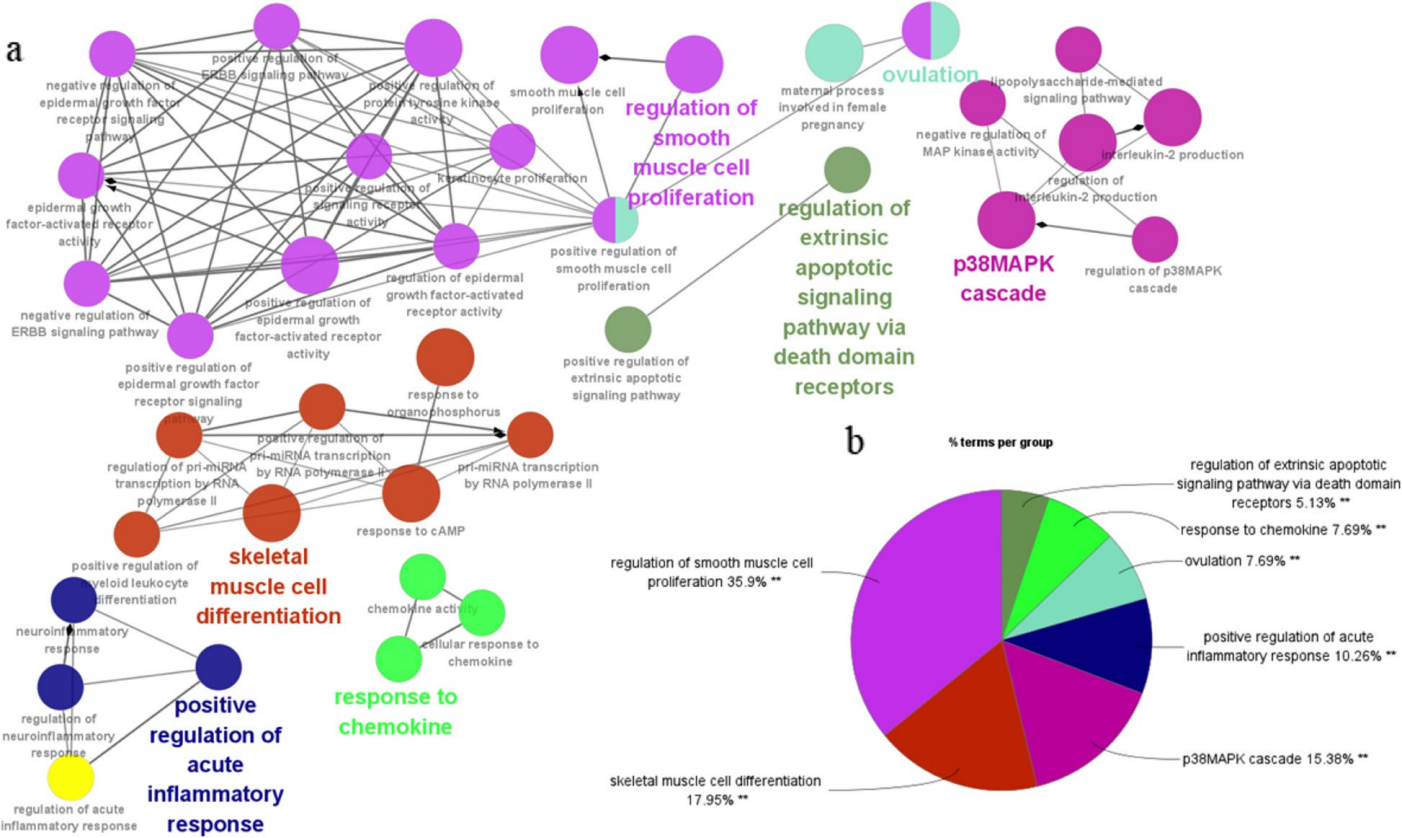
The enrichment analysis by ClueGO. **a** The network of GO terms created by the ClueGO, in which the significant term of each group is highlighted. **b** The proportion of each group in total GO terms. GO, gene ontology. **p < 0.05

Forty genes were overlapped in positively related modules between SLE and IPF, which were defined as gene set 1 (Fig. 2). These genes were highly related to pathogenesis of both SLE and IPF. GO enrichment analysis was performed with GlueGO to explore the potential function of these common genes. The significantly enriched GO terms about biological processes were presented in Fig. 3. The top 3 were “regulation of smooth muscle cell proliferation”, “skeletal muscle cell differentiation” and “p38MAPK cascade”, accounting for 35.9, 17.95 and 15.38%, respectively. Since we tried to find a specific pathway which regulated the process between SLE and IPF and considered the key inflammation signatures between these two diseases, we selected out and focused on the inflammation related p38MAPK pathway.

### Identification and analysis of common miRNAs in SLE and IPF

**Fig. 4:**
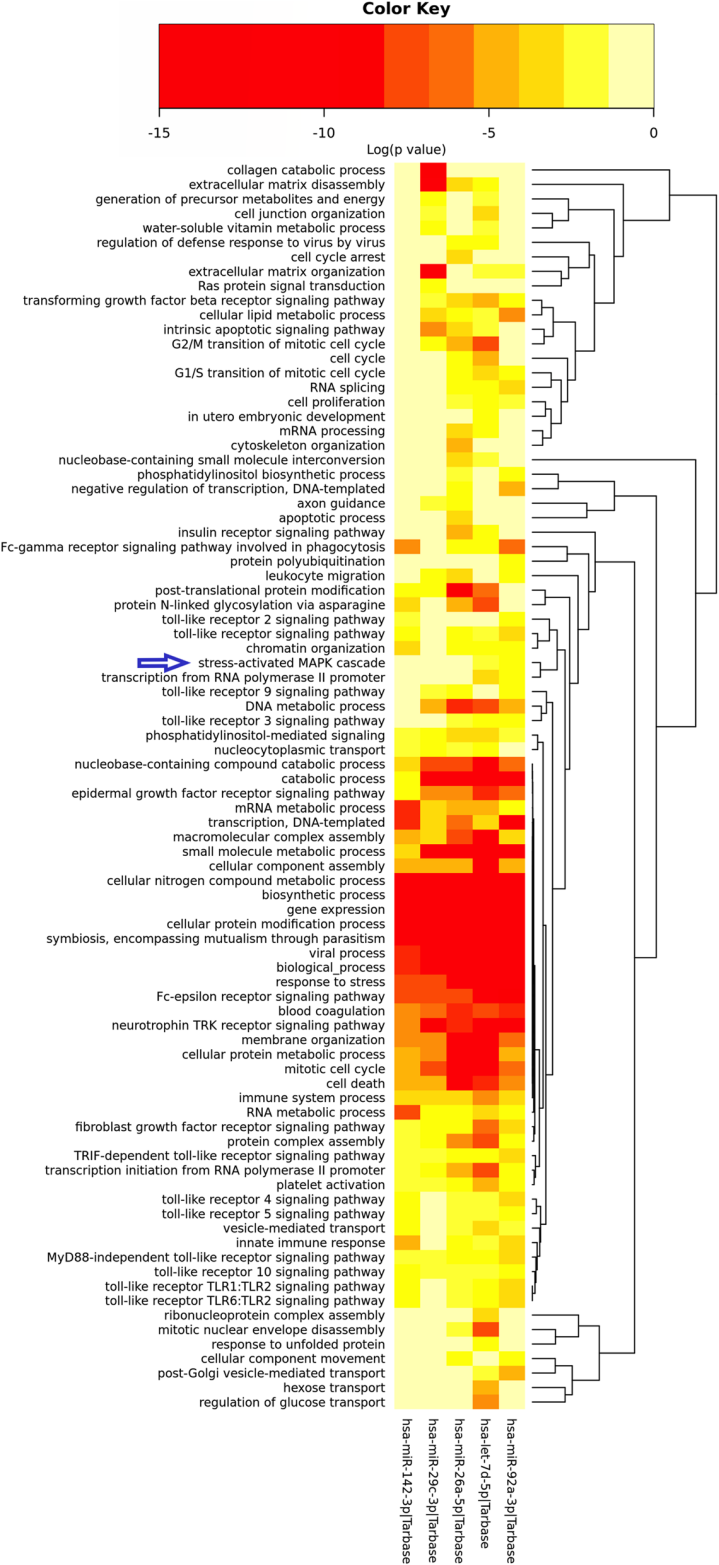
The functional enrichment analysis of the five common miRNAs by DIANA tool. The stress-activated MAPK cascade pathway was indicated by a blue arrow

Because miRNA was a vital gene expression regulatory molecule linked to various cellular activities and diseases, we tried to identify if the common pathway and genes were regulated by miRNA [21]. Totally, there were 87 miRNAs associated with SLE while 13 miRNAs associated with IPF in HMDD database. Nine common miRNAs were identified between SLE and IPF. According to previous studies, the common miRNAs which had the same disorder type of change in both SLE and IPF were selected for further research. Four common miRNAs (hsa-let-7d-5p, hsa-mir-26a-5p, hsa-mir-29c-3p and hsa-mir-92a-3p) were downregulated while 1 (hsa-mir-142-3p) was upregulated. “Cellular nitrogen compound metabolic process”, “Biosynthetic process”, “Gene expression”, “Cellular protein modification process”, “Symbiosis, encompassing mutualism through parasitism” were significantly associated with these 5 miRNAs (Fig. 4). More importantly, the heatmap showed some biological processes related to immunity and inflammation, such as “Viral process”, “Response to stress” and “Fc-epsilon receptor signaling pathway”, also had strong association with all these 5 miRNAs, indicating that the immunity and inflammation may be involved in the process of SLE and IPF. Due to the “Stress-activated MAPK cascade” enriched by the analysis, the common miRNAs between SLE and IPF may regulate the genes of MAPK cascade, especially p38MAPK.

### The gene set enrichment analysis of validated cohort in SLE and IPF

**Fig. 5:**
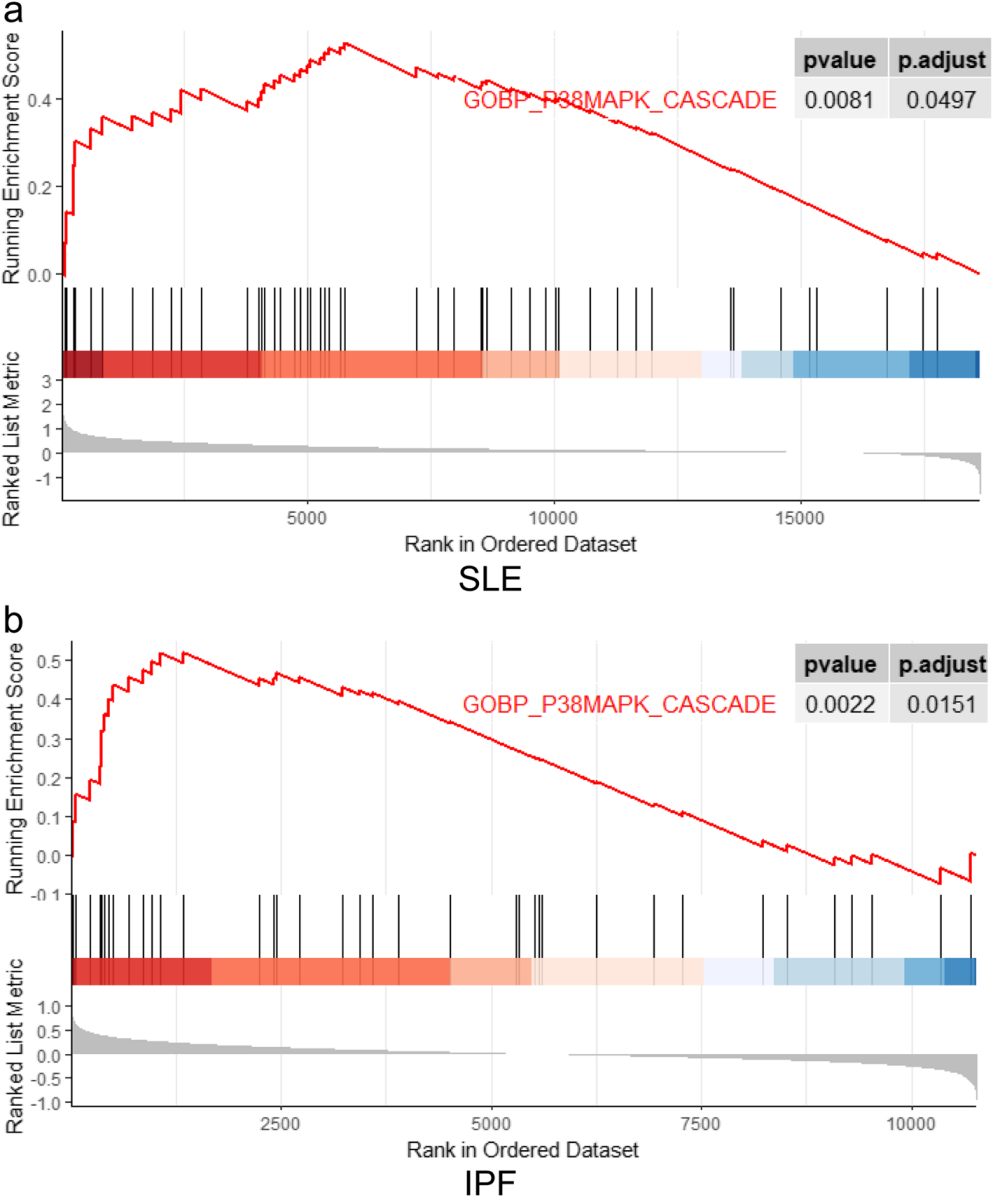
GSEA for differentially expressed gene between patients and controls. **a** The enriched result of p38MAPK cascade in GO biological progress collection in SLE. **b** The enriched result of p38MAPK cascade in GO biological progress collection in IPF

To validate the importance of p38MAPK cascade in the common pathogenesis of SLE and IPF, we performed the Gene Set Enrichment Analysis (GSEA) between patients and controls in the GSE154851 and GSE33566 datasets. The GSEAs of SLE and IPF were perfumed with GO biological process. As shown in figure (Fig. 5), activated p38MAPK cascade GO biological process were found in both SLE and IPF (*p* = 0.0081, p.adjust = 0.0497; *p* = 0.0022,p.adjust = 0.0151), which demonstrated p38MAPK cascade played a key role in the common pathogenesis.

### The common miRNAs-shared genes network in SLE and IPF

To further analysis of 5 common miRNAs, this network was constructed through TargetScan database (Fig. S1). The five miRNAs, represented by green triangle, had 4941 predicted target genes in total, in which 12 target genes that marked in blue were found in the SLE and IPF common gene set 1. The network was constructed with 17 nodes (5 miRNAs and 12 genes) and 19 edges. Moreover, these target genes which were included in the HALLMARK_HYPOXIA gene set were marked in yellow. The genes in this gene set were up-regulated in response to low oxygen level, which indicated that these genes were associated with the process of the pulmonary fibrosis.

### PPI network and molecular docking in SLE and IPF

**Fig. 6:**
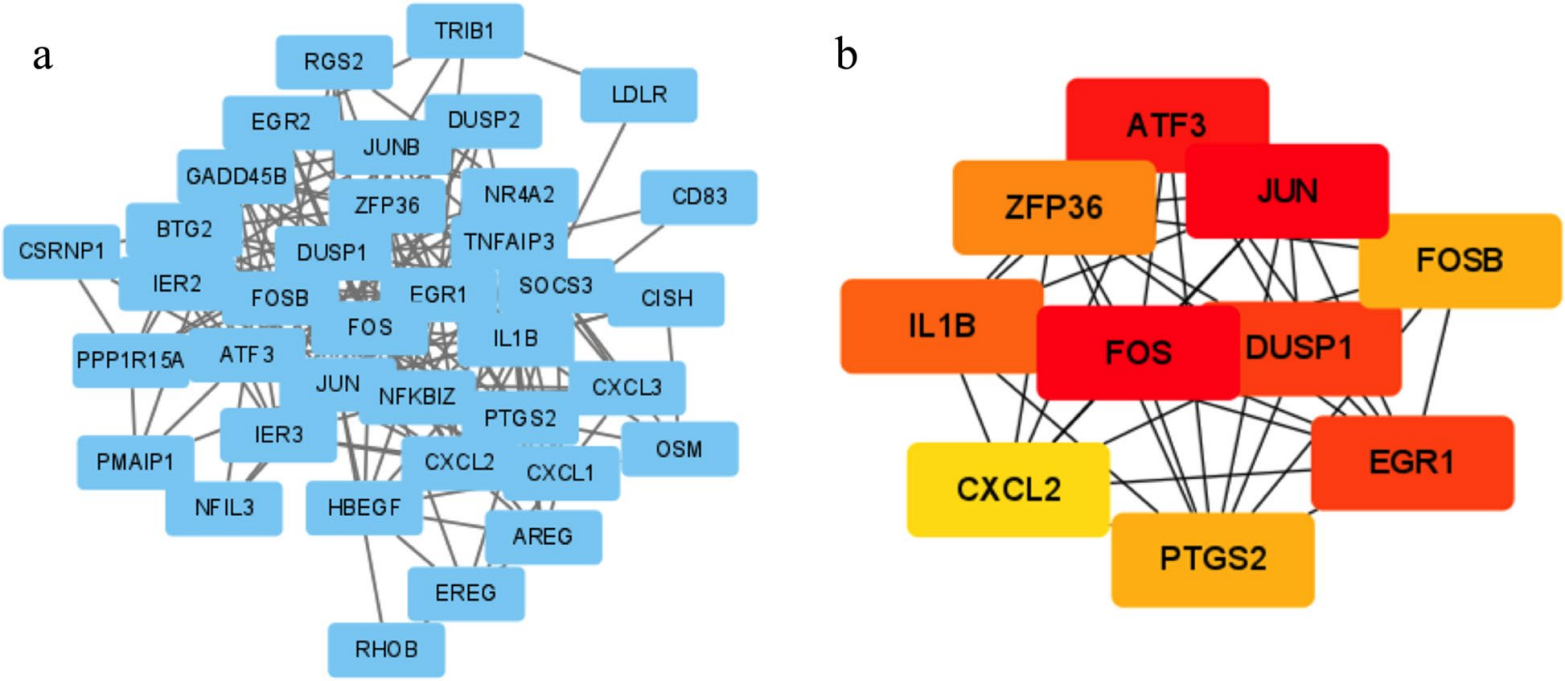
The protein-protein interaction network and the top10 genes of shared gene between SLE and IPF. **a** The protein-protein interaction network of shared genes. **b** The TOP10 genes which have the most edges. Deeper color means more edges

**Fig. 7:**
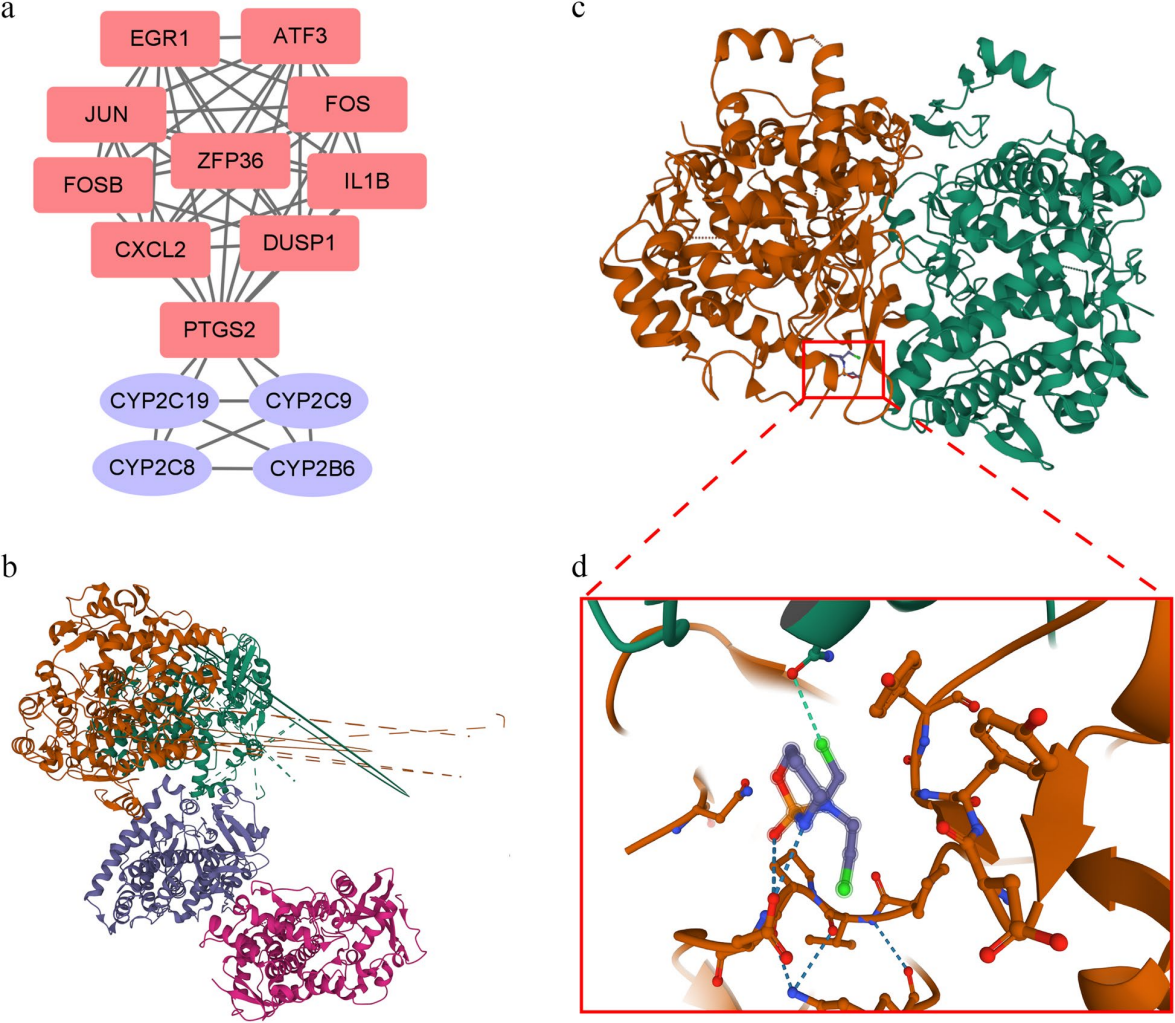
The protein-protein interaction network between target genes of cyclophosphamide and top10 genes and the possible pose of molecular docking. **a** The blue ellipses represent the target genes of cyclophosphamide while the red rectangles represent the top10 genes between SLE and IPF. **b** A possible docking between PTGS2 and CYP2C19, green and orange representing PTGS2, blue and pink representing CYP2C19. **c** A possible docking between PTGS2 and cyclophosphamide. **d** The cyclophosphamide and surrounding forces in docking

The PPI network included 40 common genes between SLE and IPF, and top 10 genes were selected (Fig. 6). Cyclophosphamide is a typical drug to treat SLE and related symptoms. The target genes of cyclophosphamide were selected and the interaction of the drug targets and top 10 genes was shown in the PPI network (Fig. 7a). Meanwhile, we used molecular docking to evaluate the possibility of affinity of PTGS2 and CYP2C19, one of protein encoded by the target genes of cyclophosphamide, the ZDOCK Score was used and their best pose interaction were calculated. The highest ZDOCK Score of PTGS2 and CYP2C19 was 2216.553 (Fig. 7b). Meanwhile, the possibility of interaction of PTGS2 and cyclophosphamide was demonstrated with Autodock Vina v.1.2.2. and the lowest binding energy was − 4.211 kcal/mol (Fig. 7c, d).

### Infiltrating immune cell score in SLE and IPF

**Fig. 8:**
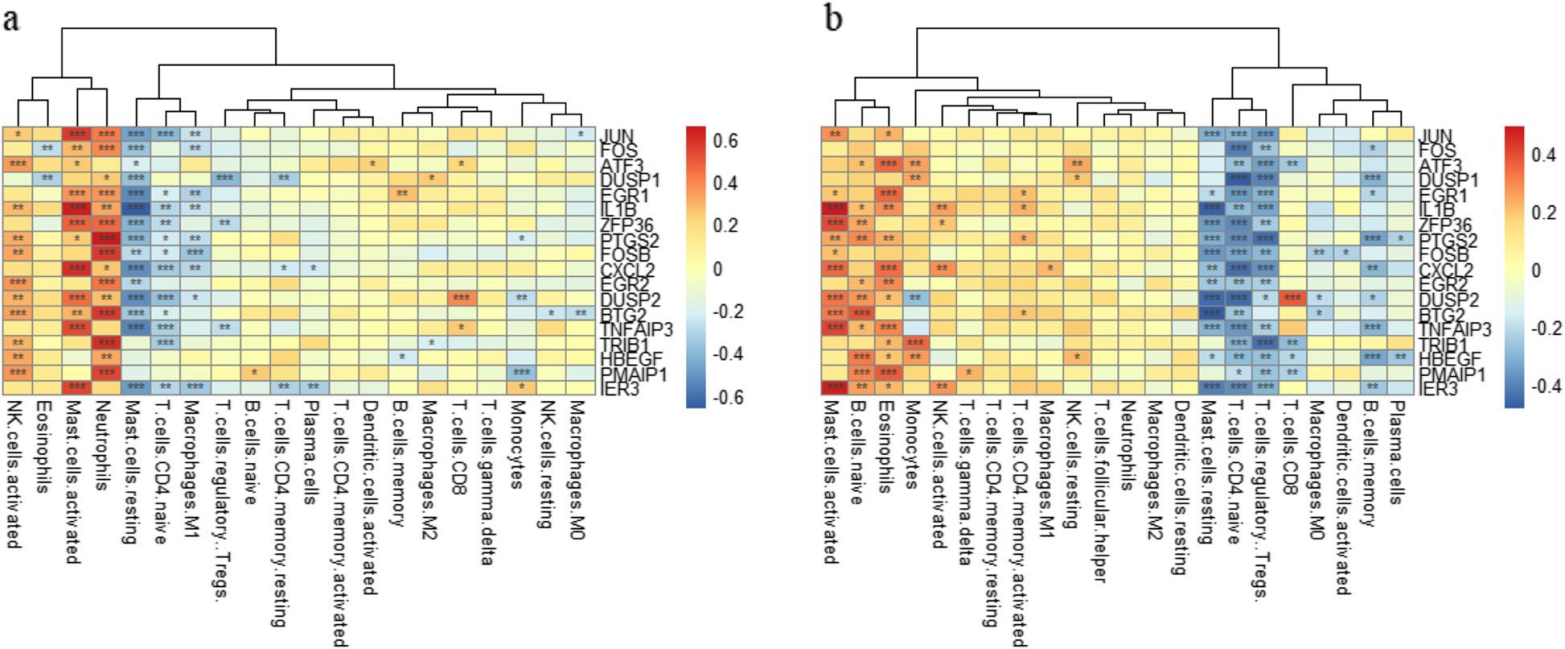
The state of infiltrating Immune cell in SLE and IPF. **a** Infiltrating immune cell score of the shared genes regulated by the 5 common miRNAs and the TOP 10 genes in SLE. **b** Infiltrating Immune cell score of the shared genes regulated by the 5 common miRNAs and the TOP 10 genes in IPF. *p < 0.1, **p < 0.05, ***p < 0.01

The shared genes which were regulated by the 5 common miRNAs were also selected for further studies. According to the score of CIBERSORT, these shared genes and top 10 genes had a strong association with many kinds of the infiltrating immune cells, demonstrating that the immune system may involve in the process of SLE and IPF. Decreased Tregs, naïve CD4+ T cells and rest mast cells but increased activated NK cells and activated mast cells were observed in both SLE and IPF (Fig. 8).

## Discussion

SLE is thought as a typical systematic autoimmune disease, leading to various symptoms in multiple organs [22]. The abnormal autoantibody or immune complex in SLE can result in the extensive inflammation and fibrosis. Although it is an infrequent complication, SLE may cause pulmonary fibrosis. Research suggested the pulmonary arterial hypertension with poor prognosis in SLE patients, one clinical feature of pulmonary fibrosis, could occur due to interstitial pulmonary fibrosis after SLE [23]. The continue development of pulmonary fibrosis even leads to ventilatory disorder and damaged heart function. Previous studies have partly demonstrated the relationship between SLE and pulmonary fibrosis. Agnieszka et al. suggested that CXCL10 and CXCL11 were the potential factors of pulmonary fibrosis in SLE patients because they found these two chemokines could cause neutrophils accumulation in the alveolar space [9]. SLE-derived pulmonary fibrosis can be treated by cyclophosphamide and glucocorticoid, and now more and more drugs to inhibit immune response were used in SLE-derived pulmonary fibrosis. Although some mechanisms have been come up with, the association between SLE and pulmonary fibrosis is still unclear. We tried to find the pathogenesis and therapeutic targets of SLE-derived pulmonary fibrosis.

Inflammation played an important role between SLE and IPF and previous studies have shown their close association [24, 25]. The biological process analysis of common miRNAs in both SLE and IPF found out “cellular nitrogen compound metabolic process”, “biosynthetic process”, “gene expression”, “cellular protein modification process”, “symbiosis, encompassing mutualism through parasitism” were significantly regulated by all these common miRNAs. Interestingly, some of common miRNAs participated in the regulation of “stress-activated MAPK cascade”, which was one of the important inflammation pathways. Between SLE patients and healthy controls, a comparative study has indicated that MAPK was the most important signaling pathway in the SLE pathogenesis with bioinformatic analysis [26]. Yu et al. found the over-expression of TRB3 could increase the expression of MAPK signaling pathway-related protein to promote the pulmonary fibrosis in the murine model [27]. Then, the common expression genes in SLE and IPF were enriched in the GO terms “p38MAPK cascade” and validated datasets also showed the importance of this pathway. MAPK cascade, especially p38MAPK, may play an important role in the SLE-derived pulmonary fibrosis. p38MAPK could be activated by cytokines and stress. It played a central role in the inflammation and a regulatory role in the immune system [28]. Some researchers have indicated that p38MAPK signal pathway was activated in glomerular endothelial cells of lupus mice, and inhibition of p38MAPK would ameliorate the renal injury caused by SLE [29, 30]. The abnormal activated p38MAPK also caused the over-activated lymphocytes in SLE [31]. These reports suggested the extensive activated p38MAPK in SLE patients. Previous studies have shown the key role of p38MAPK in pulmonary fibrosis. Chen et al. found that p38MAPK played an important role in the TGF-β1-induced human alveolar epithelial to mesenchymal transition, which was considered as a mechanism of IPF [32]. Shen et al. used a novel compound to inhibit the activation of TGF-β1/p38MAPK pathway, leading to the attenuation of pulmonary fibrosis finally [33]. In short, the p38MAPK signaling pathway, activated by other stimulators, can lead to the pulmonary fibrosis. We suggested that SLE cause extensive inflammation, inappropriate cytokines and activation of p38MAPK signaling pathway in many tissues and organs, including lung, and leads to damage and fibrosis in lung.

In our research, the common miRNAs-regulated common genes and top 10 genes were strongly associated with the infiltrating immune cell score, which meant immunity system played a role in the pathogenesis. The genes in the network, regulated by more than one common miRNAs, were considered more important. Recently, as the center of regulation of immune system, targeting to Tregs have shown the practicability in the treatment of many disease [34], so we deeply consider the function of Tregs in the SLE-derived pulmonary fibrosis. Activated T cells can release some cytokines, such as IL-4, IL-13, to promote the fibrosis [35]. Tregs can regulate the activated T cells to inhibit immunity and inflammation response. In our research, many the top and shared-genes that were regulated by common miRNAs were negative with the infiltrating score of Tregs, especially in IPF patients. The low infiltrating score of Tregs means the decreased Tregs leading to the lack of inhibition of activated T cells. Because activated T cells take part in the process of pulmonary fibrosis through cytokines, the decreased Tregs may cause pulmonary fibrosis through over-activating T cells. Interestingly, studies suggested that lymphocytes were hyperactivated in SLE, which was related to p38MAPK [31]. This suggested that the activated p38MAPK pathway in SLE may lead to fibrosis by hyperactivated lymphocytes. However, the function of Tregs in the fibrosis is complicated. Tregs can release TGF-β to promote the formation and development of fibrosis directly [36]. So, the function of T cells, especially Tregs, needs further research in the field of pulmonary fibrosis. According to the result of the immune-cell infiltration (unshown), mast cells may also participate in the process of SLE-derived pulmonary fibrosis. The increase of FcεRIα and decrease of IgE in SLE may activate the mast cells to participate in the process of SLE [37] and the mast cells could cause pulmonary fibrosis [38]. Additionally, in response of stimulation, NK cells in active SLE patients produced significantly increased IFN-γ but the role of IFN-γ and NK cells in promotion or inhibition of pulmonary fibrosis remains unclear [39, 40].

Moreover, the common miRNAs-shared gene network and molecular docking indicated potential therapeutic targets. Cyclophosphamide is a typical drug to treat SLE with the inhibition of T and B lymphocytes and it is also used to treat lupus nephritis [41, 42]. And it was also used to treat the SLE patients with pulmonary manifestations [6]. Our study demonstrated the possibility of some target genes of cyclophosphamide and cyclophosphamide itself interacted with the PTGS2, and the possible docking poses were shown. PTGS2, also known as COX2, is an important inflammation regulator. Pro-inflammatory mediators introduce the transcription of COX2 partly through p38MAPK [43]. So PTGS2 may be a key treatment target and cyclophosphamide may have the treatment effect to the SLE-derived pulmonary fibrosis.

DUSP1, also known as MKP-1, could dephosphorylation of p38MAPK to suppress inflammation [44]. It displayed significantly negative correlation with the infiltrating score of Tregs in both SLE and IPF. It has been proved that DUSP1 is required for adaptive immunity and for T cell activation and function [45]. Target to it may normalize the function of Tregs and suppress the inappropriate activation of T cells. The shared gene HBEGF contributes to the modulating airway fibrosis and pulmonary epithelial-mesenchymal transition [46] and inhibition of it may control the airway remodeling and inflammation [47]. PMAIP1 is associated with the radiation-induced pulmonary fibrosis [48]. The shared gene TNFAIP3 is associated with airway inflammatory responses although it is a negative regulator of inflammation [49] These results indicated the potential function of shared genes which regulated by common miRNAs in the SLE-derived pulmonary fibrosis.

As the symptoms appeared, SLE patients may complicate pulmonary fibrosis, even IPF. However, the treatment of SLE-derived pulmonary fibrosis is limited, and the mechanism remains unclear. This study demonstrates the potential mechanism of SLE-derived pulmonary fibrosis and point out the potential treatment target PTGS2 of cyclophosphamide. However, due to the tiny sample-size of patients with both SLE and IPF, further validations are needed in the future.

## Conclusions

Pulmonary fibrosis is a severe complication of SLE, which could cause poor prognosis of SLE patients. The infiltration of immune-cell subsets participates in this process. We found that significantly decreased Tregs, naïve CD4+ T cells and rest mast cells but increased activated NK cells and activated mast cells are common character in both SLE and IPF. In addition, the activation of MAPK cascade may be another important pathogenesis. Coincidently, the common gene PTGS2 that have a strong association with MAPK cascade is a target for cyclophosphamide which is widely used in SLE treatment. Our study reveals the potential pathogenesis of SLE-derived pulmonary fibrosis, which could give an effective clue to treat or alleviate pulmonary fibrosis in SLE patients.

## Supplementary Information

**Additional file 1: Fig. S1.** The regulatory network between miRNAs and shared genes of SLE and IPF. Green triangles represent the common miRNAs. Rectangles represent the shared gene between SLE and IPF, in which the shared gene, included in the HALLMARK_HYPOXIA gene set, marked yellow.

**Additional file 2.** microRNA in SLE.

**Additional file 3.** microRNA in IPF.

**Additional file 4.** Common genes in SLE and IPF.

## Data Availability

Data analyzed in this study is publicly available from GEO database (accession No.: GSE50772 and GSE28042; accession No.: GSE154851 and GSE33566).
