# Supplementary figures and images for: Identification of the shared genes and immune signatures between systemic lupus erythematosus and idiopathic pulmonary fibrosis
Source: Hereditas. 2023 Mar 4;160:9. doi: 10.1186/s41065-023-00270-3 (PMC9985223; doi:10.1186/s41065-023-00270-3)

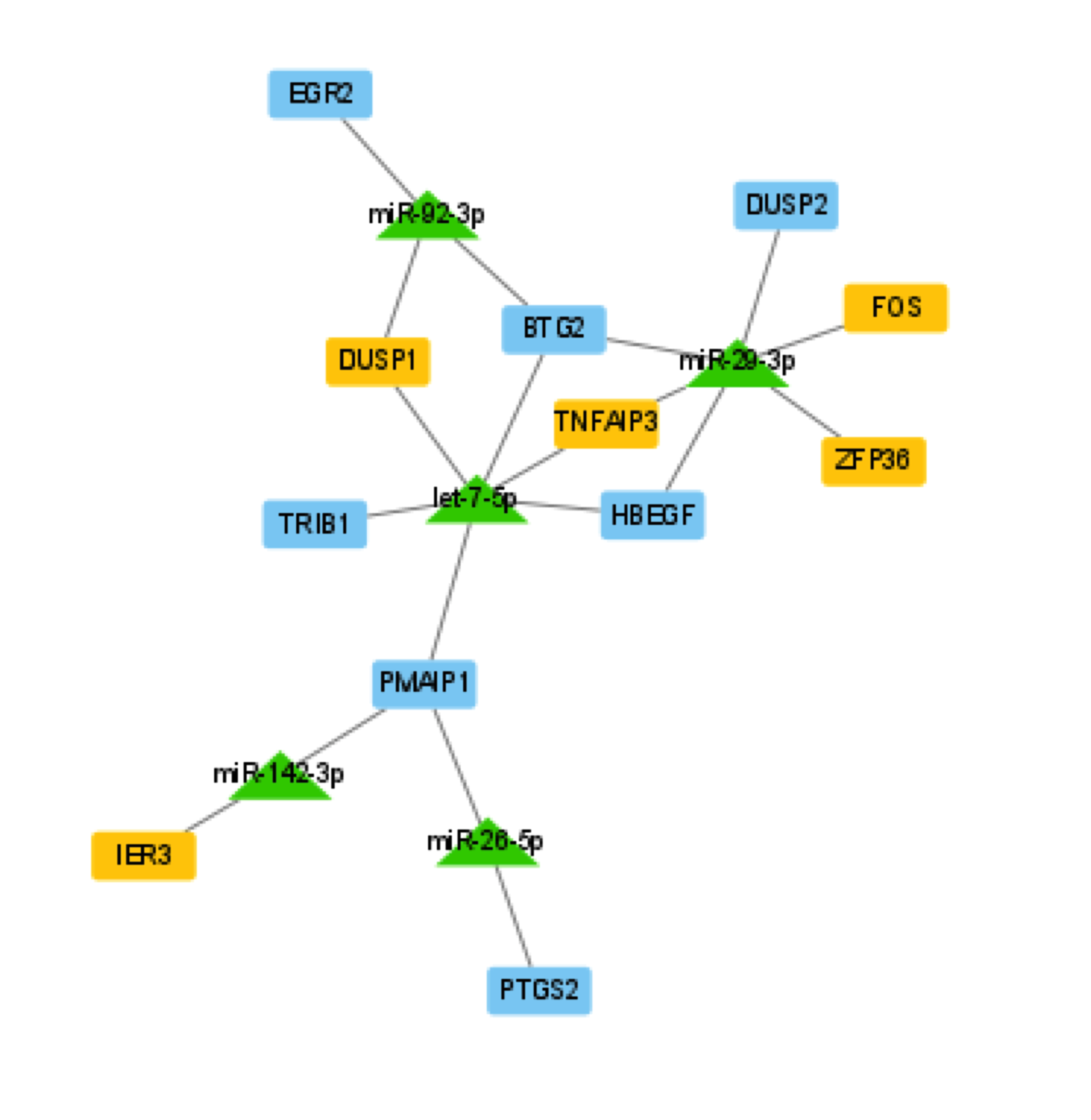

Supplement: Supplementary file 1 — Additional file 1: Fig. S1. The regulatory network between miRNAs and shared genes of SLE and IPF. Green triangles represent the common miRNAs. Rectangles represent the shared gene between SLE and IPF, in which the shared gene, included in the HALLMARK_HYPOXIA gene set, marked yellow. [file 41065_2023_270_MOESM1_ESM.tiff]
